# Triple hormone receptor agonist retatrutide for metabolic dysfunction-associated steatotic liver disease: a randomized phase 2a trial

**DOI:** 10.1038/s41591-024-03018-2

**Published:** 2024-06-10

**Authors:** Arun J. Sanyal, Lee M. Kaplan, Juan P. Frias, Bram Brouwers, Qiwei Wu, Melissa K. Thomas, Charles Harris, Nanette C. Schloot, Yu Du, Kieren J. Mather, Axel Haupt, Mark L. Hartman

**Affiliations:** 1 Stravitz-Sanyal Institute for Liver Disease and Metabolic Health and Division of Gastroenterology, Hepatology and Nutrition, Virginia Commonwealth University School of Medicine, Richmond, VA USA; 2 Section of Obesity Medicine and Weight and Wellness Center, Geisel School of Medicine at Dartmouth, Hanover, NH USA; 3 Velocity Clinical Research, Los Angeles, CA USA; 4 Eli Lilly and Company, Indianapolis, IN USA; 5 Lilly Deutschland GmbH, Bad Homburg, Germany

**Keywords:** Non-alcoholic steatohepatitis, Non-alcoholic fatty liver disease, Obesity

## Abstract

Retatrutide is a novel triple agonist of the glucose-dependent insulinotropic polypeptide, glucagon-like peptide 1 and glucagon receptors. A 48-week phase 2 obesity study demonstrated weight reductions of 22.8% and 24.2% with retatrutide 8 and 12 mg, respectively. The primary objective of this substudy was to assess mean relative change from baseline in liver fat (LF) at 24 weeks in participants from that study with metabolic dysfunction-associated steatotic liver disease and ≥10% of LF. Here, in this randomized, double-blind, placebo-controlled trial, participants (*n* = 98) were randomly assigned to 48 weeks of once-weekly subcutaneous retatrutide (1, 4, 8 or 12 mg dose) or placebo. The mean relative change from baseline in LF at 24 weeks was −42.9% (1 mg), −57.0% (4 mg), −81.4% (8 mg), −82.4% (12 mg) and +0.3% (placebo) (all *P* < 0.001 versus placebo). At 24 weeks, normal LF (<5%) was achieved by 27% (1 mg), 52% (4 mg), 79% (8 mg), 86% (12 mg) and 0% (placebo) of participants. LF reductions were significantly related to changes in body weight, abdominal fat and metabolic measures associated with improved insulin sensitivity and lipid metabolism. The ClinicalTrials.gov registration is NCT04881760.

## Abstract (web summary)

Phase 2 clinical trial results show that retatrutide, a triple agonist of the GIP, GLP-1 and glucagon receptors, results in up to 82% reduction in liver fat.

## Main

Nonalcoholic fatty liver disease, now termed metabolic dysfunction-associated steatotic liver disease (MASLD), is one of the most common chronic liver diseases in the world^1,2^. The global prevalence of MASLD has increased dramatically, from 25% in 1990–2006 to 38% in 2016–2019. Meta-regression analyses have demonstrated that the increased prevalence of obesity is a major contributor to the growing burden of MASLD^2^. At least half of patients with MASLD are estimated to have obesity^3^.

Insulin resistance is an important pathophysiologic driver for MASLD. Insulin resistance in adipocytes contributes to dysregulated lipolysis, resulting in excessive delivery of fatty acids to the liver. Substrate overload can drive hepatic de novo lipogenesis. Hepatic steatosis can trigger inflammation causing hepatocyte injury, apoptosis and necrosis, eventually leading to liver fibrosis, although numerous factors contribute to the heterogeneity of disease progression^4^. These features characterize the more progressive form of MASLD, which is called nonalcoholic steatohepatitis or metabolic dysfunction-associated steatohepatitis (MASH)^1^. Over the course of several years, worsening fibrosis may progress to cirrhosis, the development of portal hypertension and ultimately hepatic decompensation. By 2019, MASH became the second most common indication for liver transplant in the United States and was the fastest increasing indication^5^.

Currently, no treatments for MASH are approved in the United States or Europe. Many potential pharmacological therapies are in clinical development, harnessing several different mechanisms of action. Among these are the incretin-based therapies that target glucagon-like peptide 1 (GLP-1) and glucose-dependent insulinotropic polypeptide (GIP) receptors and induce weight loss. Semaglutide, a GLP-1 receptor mono-agonist, is in phase 3 development for noncirrhotic MASH, having demonstrated histological improvements in a phase 2 MASH trial^6^. The GIP/GLP-1 dual agonist, tirzepatide, reduced liver fat and improved biomarkers of MASH and fibrosis in patients with type 2 diabetes (T2D); a phase 2 trial in MASH is ongoing^7,8^. The addition of glucagon (GCG) agonist activity to GLP-1 agonism has shown promise for providing greater reduction of hepatic fat, an early marker of improvement in MASH. Efocipegtrutide (HM15211), a triple GLP-1/GIP/GCG receptor agonist, has demonstrated significant liver fat reduction after 12 weeks in participants with MASLD and is now in phase 2 development^9,10^.

Retatrutide (RETA; LY3437943) is a single protein conjugated to a fatty diacid moiety that activates human GIP, GLP-1 and GCG receptors. On the basis of cell culture studies, retatrutide is less potent than endogenous ligands of the human GCG and GLP-1 receptors (0.3 and 0.4 times as active, respectively) and is more potent at the human GIP receptor (by a factor of 8.9)^11^. The pharmacokinetics of retatrutide are dose proportional; it has a half-life of approximately 6 days, enabling weekly subcutaneous administration^12^. In a phase 2 study of retatrutide in people with obesity who did not have T2D, a weight reduction up to 24.2% was observed after 48 weeks. Treatment was also associated with improvements in blood pressure (BP), lipids and glycemia^13^. In this Article, we report the results of a substudy of that trial that evaluated changes in liver fat and biomarkers of MASH and fibrosis in people with MASLD.

## Results

### Patient disposition

**Extended Data Fig. 1:**
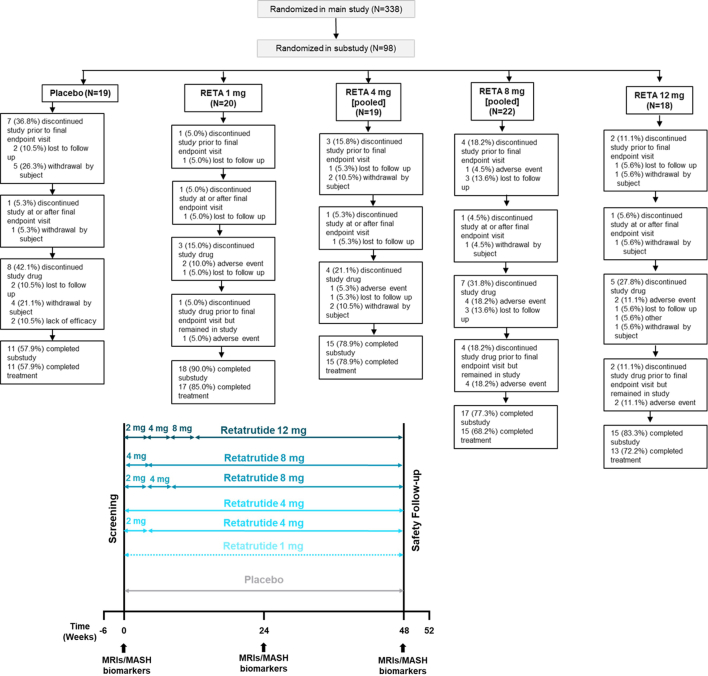
Study design and trial disposition. Top: patient disposition (CONSORT diagram) of the substudy. Completion of the substudy refers to completing the study visits, including the safety follow-up period, irrespective of adherence to study treatment. Participants who completed treatment stayed on study treatment for the treatment period. Bottom: study design schema with MRI/MASH biomarker schedule. Where applicable, dose escalations occurred every 4 weeks.

From 20 May 2021 to 22 November 2022, 498 participants were screened and 338 participants were randomized in the main obesity study. Of these 338, 98 (29.1%) met the inclusion criterion of 10% or greater liver fat content by magnetic resonance imaging proton density fat fraction (MRI–PDFF) for participation in the MASLD substudy. Participants were randomized to placebo (PBO; *n* = 19) or retatrutide 1 mg (*n* = 20), 4 mg (*n* = 19), 8 mg (*n* = 22) or 12 mg (*n* = 18) administered once weekly. The substudy was completed by 11 participants (58%) in the placebo group and by 18 (90%), 15 (80%), 17 (77%) and 15 (83%) participants in the retatrutide 1 mg, 4 mg, 8 mg and 12 mg groups, respectively. The efficacy and safety analysis population for the substudy included 98 participants. Overall, 76 (77.6%) participants completed the substudy and 71 (72.4%) participants completed the substudy on treatment (Extended Data Fig. 1). Fewer participants had MRIs with liver fat data available at week 48 (43.9%) than at week 24 (78.6%) due to early treatment discontinuation or imaging assessments being performed outside the 48 week visit window.

### Baseline demographics and clinical characteristics

The baseline demographics and clinical characteristics (Table 1) were similar across treatment groups. Overall, 46 (46.9%) participants were female, 98.0% were white and 41.8% identified as Hispanic or Latino. At baseline, participants had a mean age of 46.6 years, weight of 110.2 kg and body mass index (BMI) of 38.4 kg m^−^^2^. The baseline demographics were comparable between the main study and the MASLD substudy; however, the MASLD subset had a slightly higher mean BMI (38.4 kg m^−^^2^ versus 37.3 kg m^−^^2^) and a lower representation of African Americans (2% versus 8%)^13^. At baseline, mean alanine aminotransferase (ALT), aspartate aminotransferase (AST), fibrosis-4 (FIB-4) index, Enhanced Liver Fibrosis (ELF) score, cytokeratin-18 (K-18) and serum pro-peptide collagen III (pro-C3) were normal or modestly elevated (Table 2).

**Table 1:** Baseline demographics and characteristics of randomized substudy participants

	Placebo (*N* = 19)	RETA 1 mg (*N* = 20)	RETA 4 mg (*N* = 19)	RETA 8 mg (*N* = 22)	RETA 12 mg (*N* = 18)	Total (*N* = 98)	Overall *P* value^a^
**Sex,** ***n*** **(%)**							
Female	10 (52.6)	9 (45.0)	8 (42.1)	9 (40.9)	10 (55.6)	46 (46.9)	0.859
**Age (years)**							
	45.5 (10.7)	50.4 (13.1)	45.3 (10.0)	47.7 (11.6)	43.4 (14.3)	46.6 (12.0)	0.438
**Ethnicity,** ***n*** **(%)**							
Hispanic or Latino	8 (42.1)	6 (30.0)	9 (47.4)	11 (50.0)	7 (38.9)	41 (41.8)	0.725
**Race,** ***n*** **(%)**^**b**^							0.543
Asian	–	–	–	–	–	–	
Black or African American	1 (5.3)	1 (5.0)	0	0	0	2 (2.0)	
Multiple	–	–	–	–	–		
White	18 (94.7)	19 (95.0)	19 (100.0)	22 (100.0)	18 (100.0)	96 (98.0)	
**Weight (kg)**							
	110.8 (16.5)	108.3 (17.2)	110.4 (17.0)	107.9 (20.3)	114.6 (22.4)	110.2 (18.6)	0.820
**BMI (kg** **m**^−^^**2**^**)**							
	38.6 (4.6)	38.6 (5.6)	38.3 (4.7)	37.1 (4.9)	39.7 (6.1)	38.4 (5.2)	0.627
**BMI category,** ***n*** **(%)**							
<30	0	0	0	1 (4.5)	0	1 (1.0)	0.741
≥30 to <35	5 (26.3)	8 (40.0)	6 (31.6)	6 (27.3)	5 (27.8)	30 (30.6)	
≥35 to <40	8 (42.1)	4 (20.0)	6 (31.6)	10 (45.5)	5 (27.8)	33 (33.7)	
≥40	6 (31.6)	8 (40.0)	7 (36.8)	5 (22.7)	8 (44.4)	34 (34.7)	
**Waist circumference (cm)**							
	118.3 (12.5)	118.0 (13.0)	117.4 (11.7)	115.9 (13.3)	122.7 (16.6)	118.3 (13.4)	0.605
**HbA1c, %**							
	5.56 (0.33)	5.62 (0.40)	5.63 (0.36)	5.63 (0.38)	5.45 (0.43)	5.6 (0.38)	0.556
**HbA1c >5.7%,** ***n*** **(%)**							
	6 (31.6)	7 (35.0)	8 (42.1)	7 (31.8)	3 (16.7)	31 (31.6)	0.569
**Systolic BP (mm Hg)**							
	125.2 (9.0)	127.2 (17.3)	125.9 (12.2)	125.4 (15.7)	120.5 (17.7)	124.9 (14.6)	0.699
**Diastolic BP (mm Hg)**							
	83.7 (8.9)	78.2 (9.1)	80.8 (7.5)	82.5 (11.3)	81.7 (13.7)	81.4 (10.3)	0.533
**Hepatic fat fraction**							
	15.6 (5.8)	19.5 (6.3)	18.9 (6.8)	20.9 (7.8)	20.5 (6.7)	19.1 (6.9)	0.130

Data are mean (standard deviation) unless noted. HbA1c, glycated hemoglobin.

^a^Overall *P* value is for any differences among treatment groups and was computed using chi-squared test for categorical data and analysis of variance for continuous data.

^b^*P* value has been calculated considering all races.

**Table 2:** Biomarkers

	PBO *N* = 19	RETA 1 mg *N* = 20	RETA 4 mg *N* = 19	RETA 8 mg *N* = 22	RETA 12 mg *N* = 18
**Fasting insulin**					
Baseline, mU l^−1^	18.3 (2.0)	18.9 (2. 5)	22.0 (1.9)	21.3 (1.8)	20.2 (2.9)
CFB week 24, %	−7.5 (8.1)	−31.6 (9.0)	−40.8 (6.2)	−37.3 (5.9)	−47.7 (5.8)
* P* value	–	0.058	0.001	0.002	<0.001
Liver fat correlation week 24	0.381				
* P* value	<0.001				
CFB week 48, %	−12.5 (10.2)	−37.5 (7.8)	−43.7 (9.4)	−52.6 (6.3)	−70. 9 (5.2)
* P* value		0.049	0.029	0.001	<0.001
Liver fat correlation week 48	0.612				
* P* value	<0.001				
**C-peptide**					
Baseline, µg l^−1^	2.4 (0.2)	2.6 (0.2)	2.6 (0.2)	2.9 (0.2)	2.6 (0.2)
CFB week 24, %	−5.3 (6.8)	−26.3 (6.8)	−32.8 (4.9)	−28.8 (4.9)	−29.1 (6.8)
* P* value	–	0.036	0.001	0.005	0.017
Liver fat correlation week 24	0.389				
* P* value	<0.001				
CFB week 48, %	−13.3 (4.8)	−32.4 (5.8)	−33.4 (8.7)	−39.2 (4.9)	−50.5 (5.7)
* P* value		0.019	0.056	<0.001	<0.001
Liver fat correlation week 48	0.514				
* P* value	<0.001				
**HOMA2-IR (C-peptide)**					
Baseline	1.8 (0.1)	1. 9 (0.2)	2.0 (0.1)	2.1 (0.2)	1.9 (0.2)
CFB week 24, %	−6.1 (7.2)	−28.3 (6.8)	−34.8 (6.1)	−30.9 (5.5)	−33.7 (6.4)
* P* value	–	0.030	0.002	0.006	0.005
Liver fat correlation week 24	0.404				
* P* value	<0.001				
CFB week 48, %	−16.2 (5.6)	−33.7 (6.3)	−26.3 (7.5)	−42.3 (4.6)	−54.5 (5.9)
* P* value	–	0.051	0.278	<0.001	<0.001
Liver fat correlation week 48	0.510				
* P* value	<0.001				
**HOMA2-IR (insulin)**					
Baseline	2.4 (0.3)	2.4 (0.3)	2.7 (0.2)	2.7 (0.2)	2.3 (0.3)
% CFB week 24	−6.7 (8.5)	−30.5 (8.6)	−43.9 (5.3)	−35.8 (5.7)	−48.9 (5.8)
* P* value	–	0.056	<0.001	0.003	<0.001
Liver fat correlation week 24	0.381				
* P* value	0.001				
CFB week 48, %	−11.1 (9.5)	−36.3 (8.6)	−49.4 (5.9)	−52.4 (6.8)	−69.3 (6.2)
* P* value	–	0.053	<0.001	<0.001	<0.001
Liver fat correlation week 48	0.575				
* P* value	<0.001				
**Adiponectin**					
Baseline, mg l^−1^	4.2 (0.5)	3.8 (0.4)	4.2 (0.7)	4.1 (0.5)	3.6 (0.4)
CFB week 24, %	3.4 (4.8)	15.7 (4.7)	29.8 (10.8)	33.5 (5.3)	44.0 (7.8)
* P* value	–	0.069	0.017	<0.001	<0.001
Liver fat correlation week 24	−0.568				
* P* value	<0.001				
CFB week 48, %	12.8 (8.4)	22.5 (10.5)	56.9 (13.0)	99.3 (19.6)	59.3 (19.8)
* P* value		0.474	0.003	<0.001	0.014
Liver fat correlation week 48	−0.602				
* P* value	<0.001				
**Leptin**					
Baseline, µg l^−1^	39.4 (5.2)	24.0 (6.5)	30.6 (4.3)	31.9 (4.1)	42.4 (4.6)
CFB week 24, %	17.7 (10.6)	−8.3 (11.8)	−29.0 (8.8)	−41.4 (8.7)	−55.8 (7.8)
*P* value	–	0.121	0.001	<0.001	<0.001
Liver fat correlation week 24	0.413				
*P* value	<0.001				
CFB week 48, %	−0.003 (31.8)	−30.3 (14.9)	−41.3 (11.9)	−63.6 (8.1)	−68.0 (8.6)
*P* value	–	0.361	0.173	0.011	0.006
Liver fat correlation week 48	0.524				
*P* value	<0.001				
**Triglycerides**					
Baseline, mg dl^−1^	143.5 (14.5)	139.8 (15.7)	155.8 (15.5)	130.0 (12.1)	141.3 (17.4)
CFB week 24, %	−0.4 (4.6)	−15.8 (6.1)	−39.5 (7.2)	−35.4 (6.1)	−40.0 (4.5)
*P* value	–	0.050	<0.001	<0.001	<0.001
Liver fat correlation week 24	0.499				
*P* value	<0.001				
CFB week 48, %	−4.1 (6.3)	−21.8 (5.4)	−37.0 (6.2)	−41.1 (5.4)	−49.4 (5.7)
*P* value	–	0.030	<0.001	<0.001	<0.001
Liver fat correlation week 48	0.432				
*P* value	0.004				
**β-Hydroxybutyrate**					
Baseline, mg dl^−1^	1.1 (0.1)	1.0 (0.1)	1.0 (0.1)	1.0 (0.1)	1.3 (0.2)
CFB week 24, %	2.6 (19.5)	25.2 (17.0)	92.9 (36.6)	78.0 (32.2)	181.2 (54.5)
*P* value	–	0.399	0.023	0.037	<0.001
Liver fat correlation week 24	−0.335				
*P* value	0.003				
CFB week 48, %	−2.5 (27.1)	33.3 (20.7)	30.3 (25.7)	46.8 (26.2)	160.5 (46.3)
*P* value	–	0.336	0.414	0.225	0.003
Liver fat correlation week 48	−0.238				
*P* value	0.129				
**FGF21**					
Baseline, ng l^−1^	309.4 (48.6)	431.3 (62.7)	361.3 (73.8)	384.1 (47.4)	274.2 (36.8)
CFB week 24, %	−17.2 (11.5)	−26.5 (10.4)	−52.2 (9.4)	−65.7 (4.8)	−54.2 (10.2)
*P* value	–	0.573	0.024	<0.001	0.021
Liver fat correlation week 24	0.473				
*P* value	<0.001				
CFB week 48, %	9.7 (16.5)	−26.7 (18.7)	−46.3 (14.6)	−51.1 (13.1)	−38.7 (19.9)
*P* value		0.172	0.022	0.008	0.105
Liver fat correlation week 48	0.485				
*P* value	0.001				
**Free fatty acids**					
Baseline, mEq l^−1^	0.6 (0.1)	0.5 (0.1)	0.5 (0.04)	0.5 (0.05)	0.6 (0.05)
CFB week 24, %	12.6 (6.1)	−0.03 (8.0)	−0.03 (6.7)	8.6 (9.7)	1.6 (7.6)
*P* value	–	0.215	0.169	0.726	0.270
Liver fat correlation week 24	0.079				
*P* value	0.504				
CFB week 48, %	1.0 (9.9)	−12.2 (9.3)	−16.8 (11.6)	−25.2 (10.7)	7.2 (12.0)
*P* value	–	0.350	0.259	0.082	0.692
Liver fat correlation week 48	0.091				
*P* value	0.567				
**K-18**^**a**^					
Baseline, U l^−1^	275.3 (26.6)	310.9 (45.3)	263.5 (16.9)	281.1 (35.6)	305.5 (61.5)
CFB week 24, %	−18.3 (5.1)	−13.0 (12.0)	−33.2 (10.3)	−35.5 (7.4)	−27.9 (7.2)
*P* value	–	0.705	0.204	0.0478	0.296
Liver fat correlation week 24	0.232				
*P* value	0.061				
CFB week 48, %	−28.0 (6.6)	−41.0 (5.7)	−41.2 (15.3)	−49.6 (7.1)	−47.4 (5.0)
*P* value	–	0.140	0.434	0.022	0.016
Liver fat correlation week 48	0.152				
*P* value	0.361				
**Pro-C3**^**a,b**^					
Baseline, µg l^−1^	12.8 (0.6)	14.2 (1.0)	13.8 (0.9)	12.9 (0.9)	14.0 (1.2)
CFB week 24, %	−5.7 (2.5)	−11.9 (6.05)	−23.3 (3.5)	−22.7 (4.4)	−26.4 (2.3)
*P* value	–	0.359	<0.001	0.001	<0.001
Liver fat correlation week 24	0.426				
*P* value	<0.001				
CFB week 48, %	−0.3 (3.9)	−21.6 (4.6)	−21.1 (2.4)	−15.4 (4.3)	−15.5 (7.8)
*P* value	–	<0.001	<0.001	0.010	0.089
Liver fat correlation week 48	0.301				
*P* value	0.067				
**ALT**					
Baseline, IU l^−1^	31.6 (2.1)	29.1 (3.1)	35.5 (3.0)	33.9 (3.2)	34.4 (4.0)
CFB week 24, %	−13.5 (4.3)	−21.0 (6.2)	−30.3 (6.6)	−25.8 (9.0)	−21.6 (11.3)
*P* value	–	0.329	0.044	0.245	0.521
Liver fat correlation week 24	0.208				
*P* value	0.069				
CFB week 48, %	−2.9 (11.6)	−21.4 (6.0)	−33.6 (5.4)	−24.0 (8.7)	−15.8 (11.6)
*P* value	–	0.134	0.009	0.141	0.432
Liver fat correlation week 48	0.029				
*P* value	0.854				
**AST**					
Baseline, IU l^−1^	24.5 (1.2)	23.9 (1.8)	23.1 (1.2)	24.6 (1.6)	25.0 (2.1)
CFB week 24, %	−7.2 (3.2)	−13.0 (5.6)	−19.9 (5.0)	−19.3 (6.1)	−18.2 (6.9)
*P* value	–	0.380	0.042	0.096	0.164
Liver fat correlation week 24	0.241				
*P* value	0.035				
CFB week 48, %	18.3 (19.4)	−5.4 (5.6)	−21.8 (5.6)	−16.6 (6.7)	−1.5 (15.5)
*P* value	–	0.196	0.023	0.052	0.424
Liver fat correlation week 48	0.190				
*P* value	0.229				
**FIB-4 index**					
Baseline	0.7 (0.1)	0.9 (0.1)	0.7 (0.04)	0.9 (0.1)	0.7 (0.1)
CFB week 24, %	−7.2 (4.4)	−2.4 (6.1)	11.2 (8.2)	−9.4 (4.9)	−4.2 (6.5)
*P* value	–	0.535	0.052	0.746	0.698
Liver fat correlation week 24	−0.041				
*P* value	0.728				
CFB week 48, %	31.5 (33.0)	4.1 (6.0)	11.8 (11.6)	−1.2 (7.4)	25.9 (30.4)
*P* value	–	0.431	0.567	0.327	0.900
Liver fat correlation week 48	0.122				
*P* value	0.440				
**ELF test**^**a**^					
Baseline	7.8 (0.2)	8.3 (0.2)	8.2 (0.1)	8.2 (0.2)	8.0 (0.3)
CFB week 24, %	9.2 (2.5)	5.9 (2.3)	−0.6 (2.8)	4.8 (1.6)	4.5 (1.6)
*P* value	–	0.317	0.007	0.135	0.121
Liver fat correlation week 24	0.050				
*P* value	0.691				
CFB week 48, %	8.3 (2.5)	3.5 (1.5)	5.3 (3.2)	8.0 (1.8)	8.6 (2.6)
*P* value	–	0.105	0.458	0.933	0.926
Liver fat correlation week 48	−0.133				
*P* value	0.428				

Data are LSMs (s.e.m.) or geometric mean (s.e.m.) from analysis of variance. *P* values under % CFB are LSMs comparison versus PBO by using two-sided *z*-tests without multiplicity adjustment. Correlations are Spearman coefficients and *P* values. *N*, number of participants; *n*, number of observations.

^a^*n* = 89.

^b^Pro-C3 measured with the second-generation enzyme-linked immunosorbent assay (ELISA) corrected to correspond to the first-generation ELISA to enable comparisons with published literature (correction factor of 0.152).

### Changes in hepatic fat fraction

**Fig. 1:**
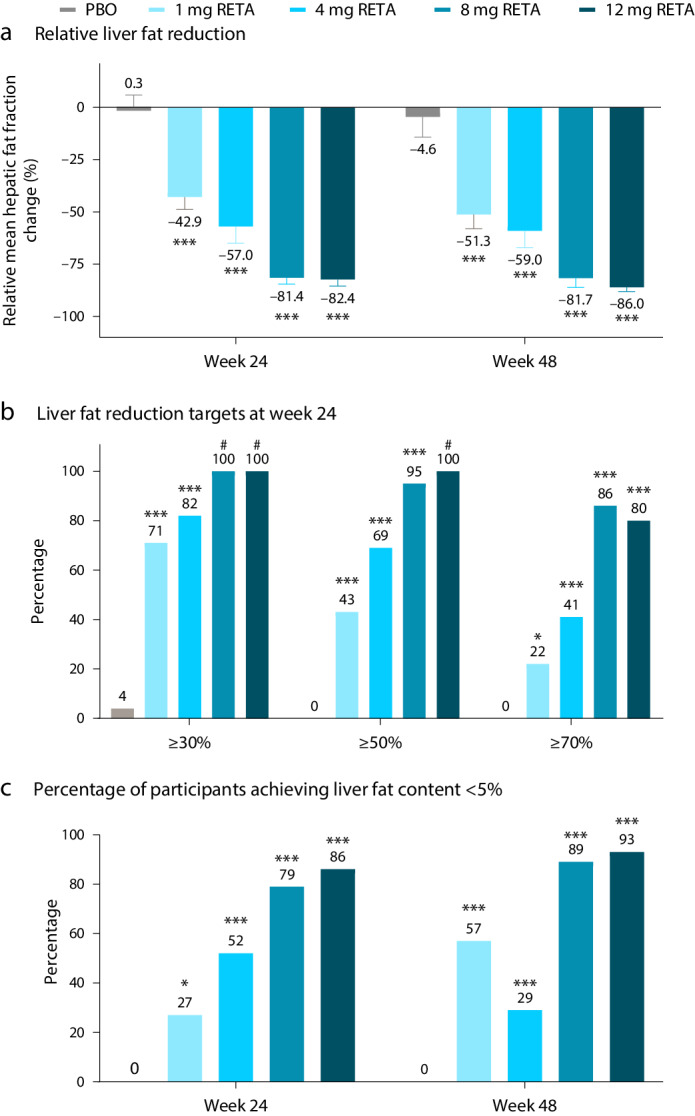
Change in liver fat. **a**, Relative liver fat reduction. Results are shown as LSM ± s.e.m. (*n* = 19 (PBO), *n* = 20 (1 mg RETA), *n* = 19 (4 mg RETA), *n* = 22 (8 mg RETA) and *n* = 18 (12 mg RETA)). **b**, The percentage of participants achieving liver fat reduction targets at week 24. **c**, The percentage of participants achieving liver fat content <5%. Comparisons versus PBO were done by using two-sided *z*-tests without multiplicity adjustment. *P < 0.05 versus PBO; ***P < 0.001 versus PBO; #, not calculable. Fewer participants had MRIs at week 48 (*n* = 8 (PBO), *n* = 9 (1 mg RETA), *n* = 9 (4 mg RETA), *n* = 8 (8 mg RETA) and *n* = 9 (12 mg RETA)) compared with week 24 (*n* = 14 (PBO), *n* = 16 (1 mg RETA), *n* = 15 (4 mg RETA), *n* = 17 (8 mg RETA) and *n* = 15 (12 mg RETA)).

**Extended Data Fig. 2:**
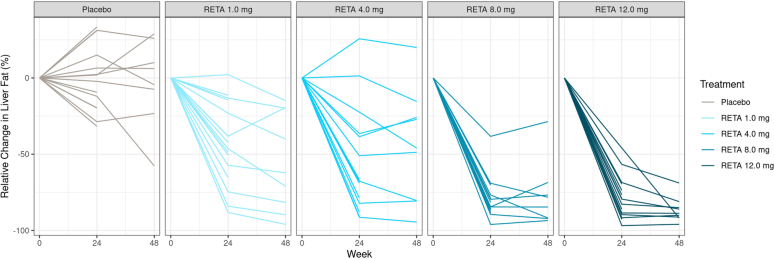
Participant-level changes in liver fat. Data for all participants is shown, excluding data after discontinuation of treatment. One of the participants in the 8 mg dose group missed the last two doses of retatrutide, despite being marked as treatment completed by the investigator. This may have contributed to the lesser relative liver fat reduction at week 48 (−68.5%) compared with week 24 (−84.5%) for this participant.

Most of the reduction in liver fat occurred within the first 24 weeks. After 24 weeks, relative liver fat reduction was significantly greater for all doses of retatrutide compared with placebo (*P* < 0.001 all doses; primary objective). The least-squares mean (LSM) relative liver fat changes from baseline with retatrutide treatment were −42.9%, −57.0%, −81.4% and −82.4% for the 1, 4, 8 and 12 mg doses, respectively, compared with +0.3% in the placebo group (Fig. 1a). The estimated treatment differences versus placebo were −43.2% (95% confidence interval −58.9 to −27.4) with 1 mg, −57.3% (−75.7 to −38.9) with 4 mg, −81.7% (−94.2 to −69.2) with 8 mg and −82.7% (−95.2 to −70.2) with 12 mg (*P* < 0.001 all doses). At 48 weeks, the relative liver fat changes from baseline with retatrutide treatment were −51.3%, −59.0%, −81.7% and −86.0% for the 1, 4, 8 and 12 mg doses, respectively, compared with −4.6% for placebo (Fig. 1a). All doses of retatrutide at week 48 were superior to placebo (*P* < 0.001 all doses); estimated treatment differences −46.7% (−70.0 to −23.4) with 1 mg, −54.4% (−79.3 to −29.5) with 4 mg, −77.1% (−98.8 to −55.4) with 8 mg and −81.4% (−101.4 to −61.4) with 12 mg. Participant-level changes in liver fat are shown in Extended Data Fig. 2.

**Extended Data Fig. 3:**
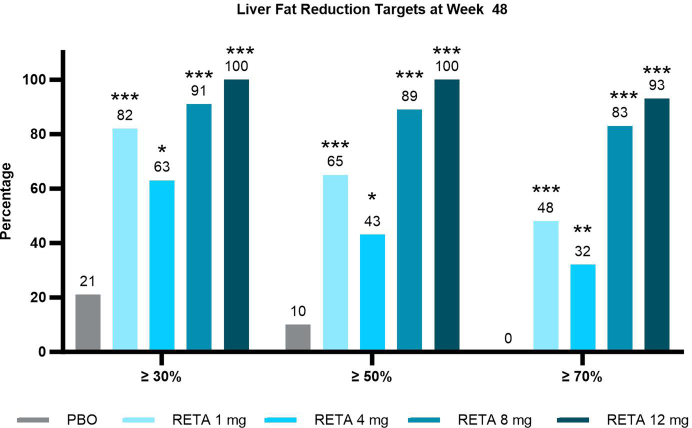
Percent of participants achieving liver fat reduction targets at week 48. Results shown as LS means ± SE. Comparisons vs. PBO were done by using 2-sided z-tests without multiplicity adjustment. Fewer participants had MRIs at Week 48 (n=8 [PBO], n=9 [1 mg RETA], n=9 [4 mg RETA], n=8 [8 mg RETA], n=9 [12 mg RETA]) compared with Week 24 (n=14 [PBO], n=16 [1 mg RETA], n=15 [4 mg RETA], n=17 [8 mg RETA], n=15 [12 mg RETA]). MRI, magnetic resonance imaging; PBO, placebo; RETA, retatrutide.

**Extended Data Fig. 4:**
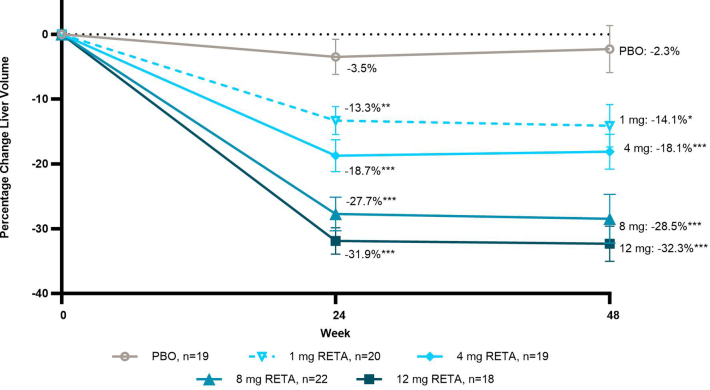
Percent change in liver volume. Results shown graphically as LS means ± 1.96 × SE. Comparisons vs. PBO were done by using 2-sided z-tests without multiplicity adjustment. LS – least square; SE – standard error; PBO – placebo; RETA – retatrutide; *p<0.05 vs. PBO; **p<0.01; ***p<0.001 vs. PBO.

At 24 weeks across the retatrutide treatment groups, 71–100% of participants achieved categorical relative liver fat reduction of 30% or more, compared with 4% in the placebo group. Liver fat reduction of 50% or greater and 70% or greater was observed in 43–100% and 22–86% of participants who received retatrutide, respectively (Fig. 1b). At 48 weeks, 63–100% of participants achieved 30% or more relative liver fat reduction compared with 21% of participants in the placebo group. Liver fat reduction of at least 50% and at least 70% was achieved by 43–100% and 32–93% of participants who received retatrutide, respectively (Extended Data Fig. 3). With the 8 mg and 12 mg doses of retatrutide, total liver fat content of <5% was achieved by 79% and 86% of participants, respectively, at week 24 and by 89% and 93%, respectively, at week 48 (Fig. 1c). In tandem with decreases in liver fat content, liver volume was significantly reduced by retatrutide compared with placebo in a dose-responsive manner by 24 weeks, and this was maintained at week 48 (Extended Data Fig. 4).

### Body weight and waist circumference

**Extended Data Fig. 5:**
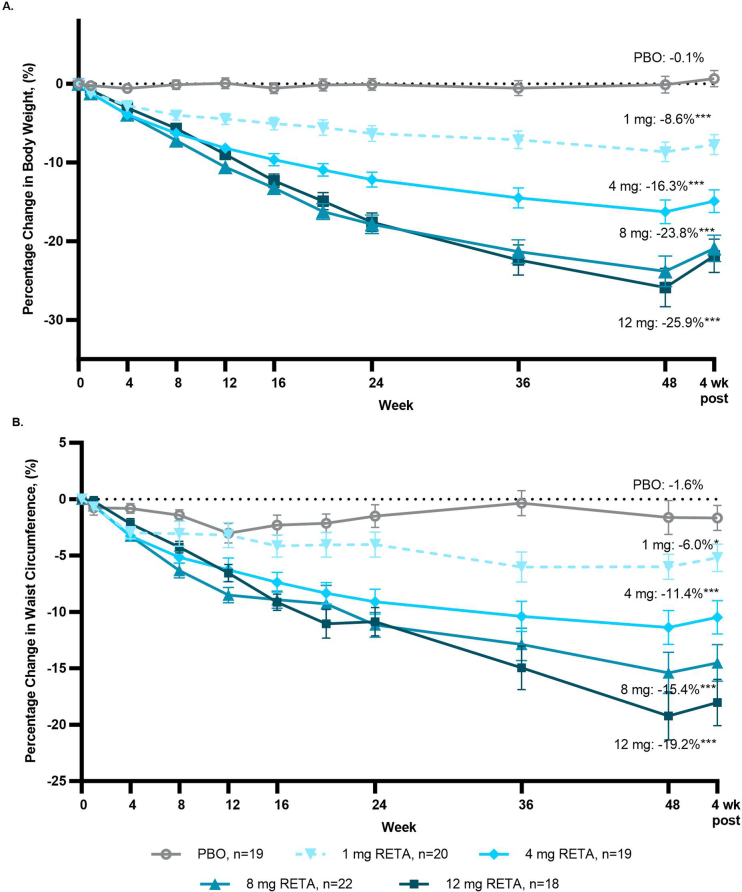
Percentage change in body weight and waist circumference. **a**, The percentage change in body weight. **b**, The percentage change in waist circumference with results shown as LS means (SE). Comparisons vs. PBO were done by using 2-sided z-tests without multiplicity adjustment. *p<0.05 vs. PBO; **p<0.01; ***p<0.001 vs. PBO. Results at the 48-week end-of-treatment visit are shown with statistical testing results. Additionally, 4-week post-treatment results are shown. LS, least squares; PBO, placebo; RETA, retatrutide; SE, standard error.

In this substudy of participants with MASLD, body weight was significantly reduced by all doses of retatrutide compared with placebo at both 24 and 48 weeks (*P* < 0.001 for all doses). The LSM (s.e.m.) percentage changes in body weight at week 24 with retatrutide treatment were −6.3% (1.0), −12.2% (0.9), −17.9% (1.2) and −17.6% (1.2) for the 1, 4, 8 and 12 mg doses, respectively, compared with −0.1% (0.7) with placebo. At 48 weeks, the LSM (s.e.m.) percentage changes in body weight were −8.6% (1.2), −16.3% (1.5), −23.8% (2.0) and −25.9% (2.4) for the 1, 4, 8 and 12 mg doses, respectively, compared with −0.1% (1.1) with placebo. Some weight regain was observed at the safety follow-up visit 4 weeks after discontinuation of treatment (Extended Data Fig. 5). Waist circumference was reduced by retatrutide treatment. At week 24, the LSM percentage change from baseline (CFB) in waist circumference ranged from −4.0% (1.1) to −11.2% (1.1) with retatrutide, compared with −1.5% (1.0) with placebo (*P* < 0.001 for all doses except 1 mg) and at week 48 the percentage change ranged from −6.0% (1.0) to −19.2% (2.1) with retatrutide treatment, compared with −1.6% (1.5) with placebo (*P* = 0.02 for 1 mg and *P* < 0.001 for all other retatrutide doses; Extended Data Fig. 5). These percent changes after 48 weeks with retatrutide treatment corresponded to mean reductions in waist circumference ranging from 6.1–20.6 cm compared with 2.5 cm with placebo. These findings are comparable to those reported in the larger main study population^13^.

**Fig. 2:**
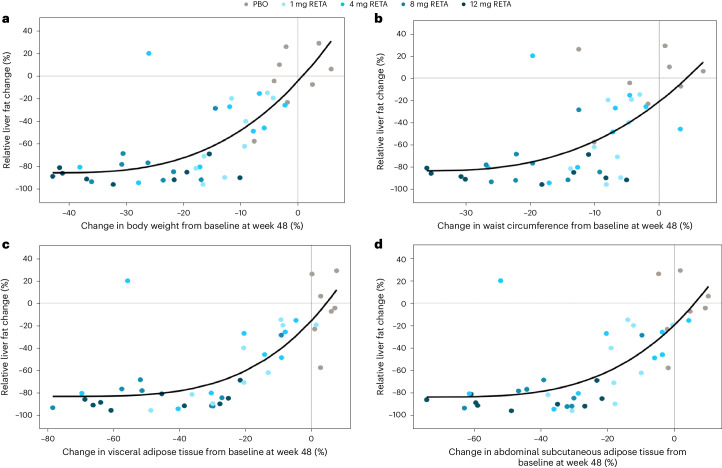
Scatter plot and fitted power model curve. **a**, Relative liver fat reduction versus percentage changes in body weight at week 48. **b**, Relative liver fat reduction versus percentage change in waist circumference at week 48. **c**, Relative liver fat reduction versus percentage change in visceral adipose tissue at week 48. **d**, Relative liver fat reduction versus percentage changes in abdominal subcutaneous adipose tissue at week 48.

Relative liver fat reduction was strongly correlated with percent change from baseline in both body weight (*r* = 0.800, *P* < 0.001) and waist circumference (*r* = 0.652, *P* < 0.001) at 24 weeks and at 48 weeks (*r* = 0.739, *p* < 0.001 for body weight and *r* = 0.601, *P* < 0.001 for waist circumference). Near-maximal reductions in liver fat were achieved coincident with approximately 20% reductions in body weight and waist circumference (Fig. 2a,b). Among retatrutide-treated participants achieving liver fat content <5%, mean BMI across the dose groups was between 31.0 and 32.9 kg m^−^^2^ at week 24 and between 25.8 and 30.8 kg m^−^^2^ at week 48 (Extended Data Table 1).

### Abdominal adipose tissue depots

**Fig. 3:**
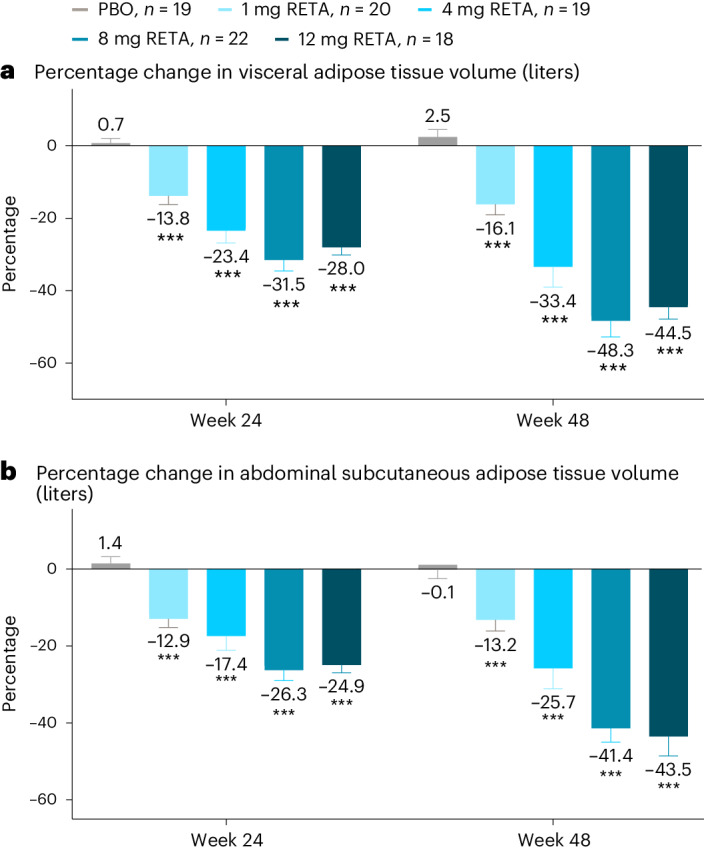
Percent change in abdominal adipose tissue depots. **a**, Percentage change in visceral adipose tissue volume (liters). **b**, Percentage change in abdominal subcutaneous adipose tissue volume (liters). Data are LSMs ± s.e.m. Comparisons versus PBO were done by using two-sided *z*-tests without multiplicity adjustment. ***P < 0.001 versus PBO.

Abdominal visceral adipose tissue (VAT) and abdominal subcutaneous adipose tissue (ASAT) were significantly reduced by all doses of retatrutide compared with placebo (*P* < 0.001 all doses). With retatrutide treatment, the percent change from baseline in VAT ranged from −13.8% (2.5) to −31.5% (3.0) at 24 weeks and from −16.1% (2.9) to −48.3% (4.5) at 48 weeks. In contrast, with placebo, VAT increased by 0.7% (1.4) at 24 weeks and by 2.5% (2.0) at 48 weeks (Fig. 3a). Similarly, reductions in ASAT with retatrutide ranged from 12.9% (2.2) to 26.3% (2.7) at 24 weeks and from 13.2% (3.0) to 43.5% (5.0) at 48 weeks compared with change of +1.4% (1.8) and −0.1% (2.4) in the placebo-treated group at weeks 24 and 48, respectively (Fig. 3b). The dose-dependencies of changes in VAT and ASAT were more evident at 48 weeks.

Relative liver fat reduction was significantly correlated with percent change from baseline in VAT (*r* = 0.792, *P* < 0.001) and ASAT (*r* = 0.742, *P* < 0.001) at 24 weeks and 48 weeks (*r* = 0.745, *P* < 0.001 for VAT and *r* = 0.703, *P* < 0.001 for ASAT). Near-maximal reductions in liver fat were achieved at approximately 40% reductions in both VAT and ASAT (Fig. 2c,d).

### Metabolic biomarkers

Treatment with retatrutide improved several markers of insulin resistance at 24 and 48 weeks, with greater changes at week 48 (Table 2). Fasting serum insulin concentrations were reduced at week 48 compared with baseline by up to 70.9% with retatrutide treatment (*P* < 0.01 versus placebo, doses 4 mg or greater). Serum C-peptide concentrations were reduced by up to 50.5% with retatrutide at 48 weeks (*P* < 0.001 versus placebo, 8 mg or greater). Significant improvements in Homeostatic Model Assessment for Insulin Resistance (HOMA2-IR) computed with fasting insulin were observed with retatrutide doses of 4 mg or greater, with changes up to −69.3% at 48 weeks (*P* < 0.001 versus placebo). Similarly, at 48 weeks HOMA2-IR computed with fasting C-peptide significantly improved with the 8 mg and 12 mg doses with changes up to −54.5% (Table 2; *P* < 0.001 versus placebo).

**Extended Data Fig. 6:**
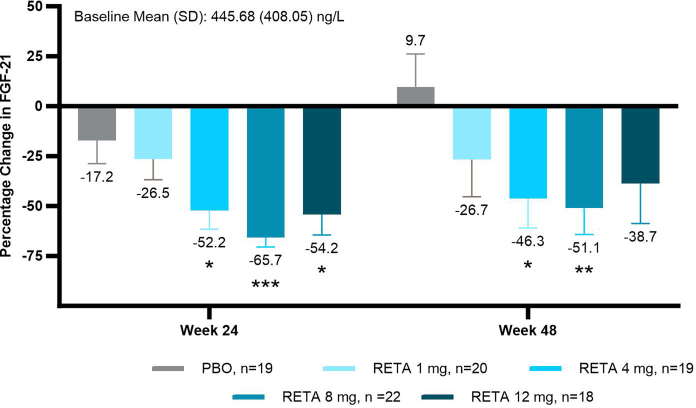
FGF21 percentage change from baseline. Results shown are LS means (SE). Comparisons vs. PBO were done by using 2-sided z-tests without multiplicity adjustment. *p<0.05 vs. PBO; **p<0.01; ***p<0.001 vs. PBO. FGF21, fibroblast growth factor 21; LS, least squares; PBO, placebo; RETA, retatrutide; SE, standard error.

Several biomarkers associated with lipid storage and metabolism were significantly changed by retatrutide treatment. Adiponectin increased significantly at weeks 24 and 48 with retatrutide 4 mg or greater (Table 2; *P* < 0.05 versus placebo). Leptin decreased significantly with retatrutide 4 mg or greater at 24 weeks (*P* < 0.01 versus placebo) and with retatrutide 8 mg or greater at 48 weeks (*P* < 0.05 versus placebo). At 24 and 48 weeks, significant reductions in fasting triglycerides were observed with retatrutide doses of 4 mg or greater (Table 2; *P* < 0.001 versus placebo). β-Hydroxybutyrate increased with retatrutide 4 mg or greater at 24 weeks (*P* < 0.05 versus placebo) and with 12 mg at 48 weeks (*P* = 0.003 versus placebo). Fibroblast growth factor 21 (FGF21) decreased with retatrutide 4 mg or greater at 24 weeks and at 48 weeks with retatrutide 4 and 8 mg (*P* < 0.05 versus placebo) (Extended Data Fig. 6). Serum-free fatty acids did not change significantly with retatrutide compared with placebo.

At 24 and 48 weeks, significant correlations were observed between relative liver fat reduction and percent change from baseline in insulin, C-peptide, HOMA2-IR (insulin), HOMA2-IR (C-peptide), triglycerides, adiponectin, leptin and FGF21 (*P* < 0.05). Relative liver fat change was significantly correlated with percent change in β-hydroxybutyrate at week 24 but not at week 48 (Table 2).

### MASH and fibrosis biomarkers

**Fig. 4:**
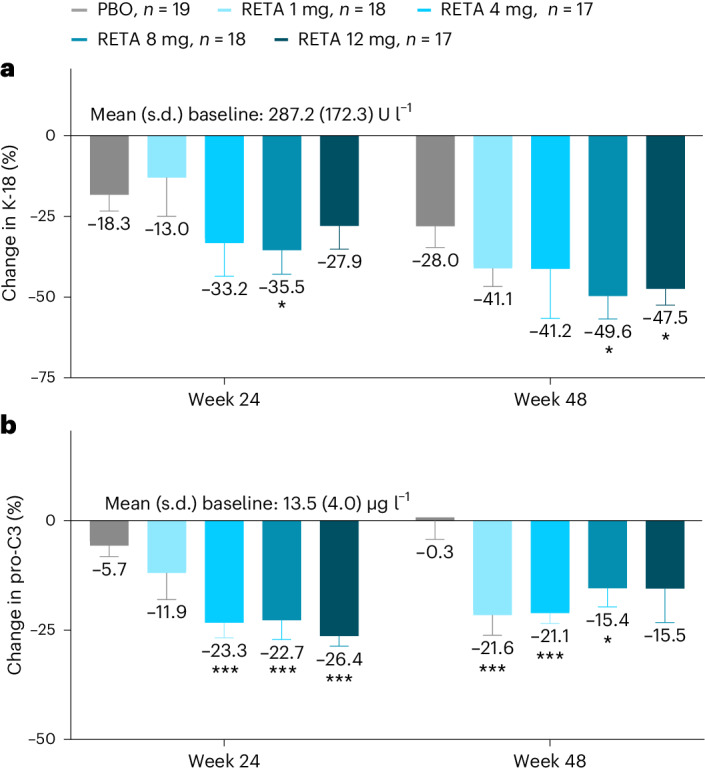
Percentage change in K-18 and pro-C3. **a**, The percentage change in K-18. **b**, The percentage change in pro-C3 with results shown as LSMs ± s.e.m. Comparisons versus PBO were done by using two-sided *z*-tests without multiplicity adjustment. *P < 0.05 versus PBO; ***P < 0.001 versus PBO. Pro-C3 measured with the second-generation ELISA corrected to correspond to the first-generation ELISA to enable comparisons to published literature (correction factor of 0.152).

**Extended Data Fig. 7:**
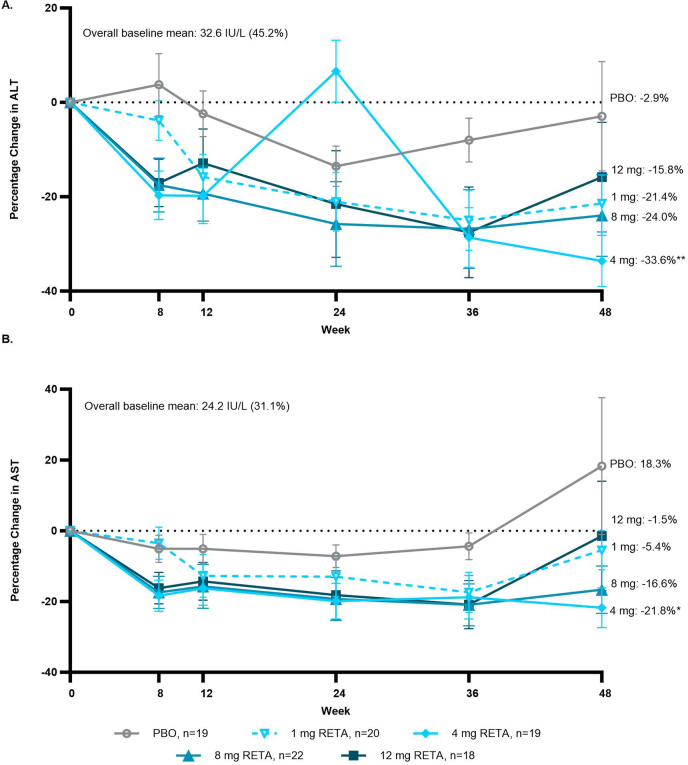
Percent change from baseline - ALT and AST. **a**, The percentage change from baseline in ALT. **b**, The percentage change from baseline in AST with results shown graphically as LS means ± 1.96 × SE; Baseline as geometric mean (Coefficient of variation%). Comparisons vs. PBO were done by using 2-sided z-tests without multiplicity adjustment. ALT=alanine aminotransferase; AST=aspartate aminotransferase; LS=least squares; PBO=placebo; RETA= retatrutide; SE=standard error.

At 24 weeks, K-18 decreased significantly with retatrutide 8 mg and at 48 weeks with retatrutide 8 and 12 mg (Table 2 and Fig. 4a; *P* < 0.05 versus placebo). Pro-C3 decreased significantly with retatrutide doses of 4 mg or greater at 24 weeks (*P* ≤ 0.001 versus placebo) and at 48 weeks with retatrutide 1 mg, 4 mg and 8 mg (Table 2 and Fig. 4b; *P* ≤ 0.01 versus placebo). Mean ALT, AST, FIB-4 and ELF did not change consistently versus placebo (Table 2 and Extended Data Fig. 7). Correlations between the change from baseline in these biomarkers and the relative change in liver fat were not statistically significant except for changes in AST and pro-C3 at 24 weeks (Table 2).

### Safety

**Extended Data Fig. 8:**
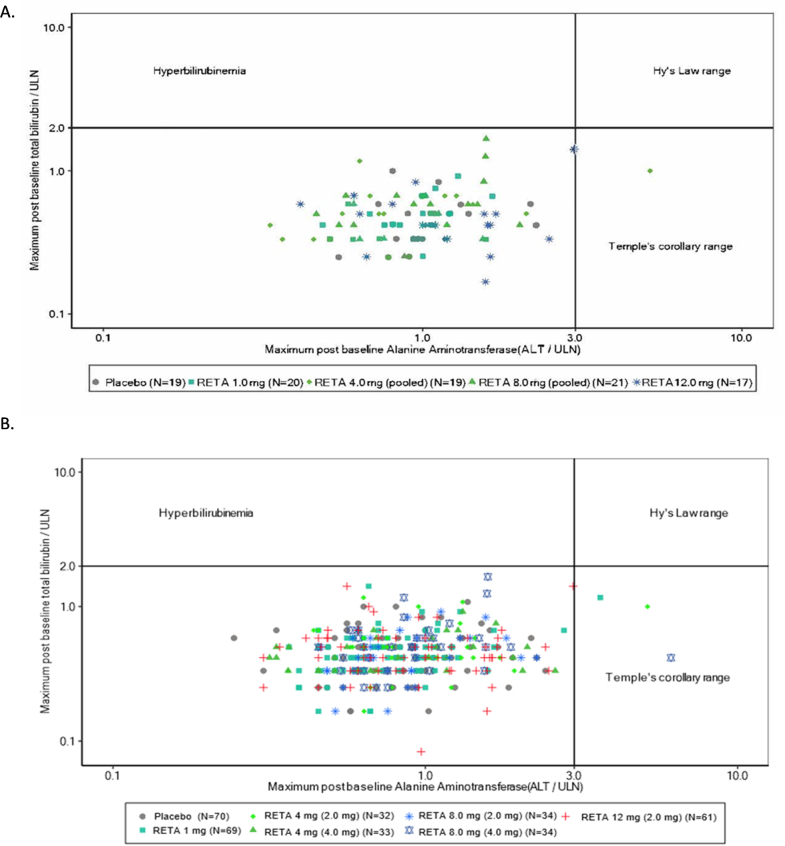
Evaluation of drug-induced serious hepatotoxicity (eDISH) plots. **a**, eDISH plot for MASLD substudy population (n=98). **b**, eDISH plot for overall obesity population (n=336). eDISH, evaluation of Drug-Induced Serious Hepatotoxicity; RETA, retatrutide; ULN, upper limit of normal. Notes on interpretation of the eDISH plot^48^. ● Hy’s Law refers to the increased risk of serious drug-induced liver injury that is present when both aminotransferase elevations (>3 times the upper limit of the normal range [ULN]) and total bilirubin elevations (>2 times the ULN) occur together. This is denoted by the upper right quadrant of the eDISH plot. ● Temple’s corollary refers to the observation that drugs which caused a marked preponderance of lesser liver injuries compared with control agents, shown by more frequent serum aminotransferase elevations, were more likely to show more Hy’s Law cases. This is denoted by the lower right quadrant of the eDISH plot. ● The upper left quadrant refers to the cholestasis range. ● The lower left quadrant includes values within or near the reference range.

The safety data for the full study population have been reported previously^13^. In this substudy of participants with MASLD, transient and generally mild-to-moderate gastrointestinal events were the most frequently reported adverse events. The frequency of these adverse events was higher in the 8 mg and 12 mg dose groups. Two participants treated with retatrutide (2.5%) experienced a total of three serious adverse events (Extended Data Table 2). There were no hepatotoxicity signals in the overall obesity trial population or in the subset of participants with MASLD through 48 weeks (Extended Data Fig. 8). Increases in β-hydroxybutyrate were not associated with ketoacidosis in any individual.

## Discussion

In this substudy of participants with MASLD who were included in a phase 2 study of people with obesity or overweight with weight-related complications, treatment for 24 weeks with the GIP/GLP-1/GCG triple receptor agonist, retatrutide, was associated with significant reductions in liver fat content at all doses. At the two highest doses, 80% or more of participants achieved ≥70% relative reduction in liver fat and more than 85% achieved resolution of steatosis, defined as <5% total liver fat content. Near-maximal liver fat reduction was achieved at an approximately 20% reduction in body weight. Reductions in liver fat were strongly associated with reductions in body weight, ASAT and VAT, and with improvements in markers of insulin sensitivity and lipid metabolism.

The 86% relative liver fat reduction observed with retatrutide 12 mg at 48 weeks is among the largest treatment effects reported so far, although differences in populations and study design across trials limit direct comparisons. In phase 1 and 2 studies in patients with MASLD or MASH, liver fat reductions have been greatest with GLP-1/GIP/GCG triple agonists, GLP-1/GCG dual agonists and FGF21 analogs. The GLP-1/GIP/GCG triple agonist, efocipegtrutide, demonstrated up to 81% liver fat reduction after 12 weeks in participants with MASLD^9^. After 24 weeks, the dual GLP-1/GCG agonists, efinopegdutide and pemvidutide, reduced liver fat by up to 73% and 76%, respectively, in participants with MASLD^14,15^. In patients with biopsy-proven MASH, efruxifermin reduced liver fat by up to 72% after 12 weeks and pegozafermin reduced liver fat by up to 48% after 24 weeks (both FGF21 analogs)^16,17^. Reported liver fat reductions with GLP-1 mono-agonists and the GIP/GLP-1 dual agonist, tirzepatide, were lower than those observed with retatrutide, ranging from 32% after 24 weeks with dulaglutide and 47% after 52 weeks with tirzepatide, to approximately 50% after 72 weeks treatment with semaglutide^8,18,19^. Rapid reductions of liver fat were reported in a study of 50 patients undergoing a very low-calorie diet for 2–3 weeks followed by bariatric surgery; mean liver fat content was reduced from a baseline of 18.1 to 14.9% after very low-calorie diet and then to 9.3%, 6.5% and 4.9% at 1, 3 and 6–10 months of postsurgical follow-up. Overall, liver fat was normalized (to <5% liver fat) in 32 (64%) of 50 participants after a mean of 22.5 weeks^20^. In the current study, more than 85% of participants treated with retatrutide 12 mg achieved normal levels of liver fat after 24 and 48 weeks. Of note, in clinical studies, a relative liver fat reduction of ≥30% has been associated with histological improvement in patients with MASH^21^. Greater reductions in liver fat may result in higher odds of histological improvement, particularly if there is complete resolution of steatosis.

The additional liver fat lowering observed with retatrutide compared with GLP-1 mono-agonists and tirzepatide may be related to the greater weight reduction achieved with retatrutide, direct hepatic effects of glucagon receptor agonism or both^13,22^. In preclinical models, retatrutide promoted weight loss by reducing food intake and increasing energy expenditure compared with calorie intake-matched animals. In addition, its glucagon activity may reduce liver fat by stimulating hepatic fatty acid oxidation and reducing hepatic lipogenesis^11,22^. In the present study, levels of β-hydroxybutyrate, a biomarker of fatty acid oxidation, increased two to threefold in a dose-related pattern with retatrutide doses 4 mg and higher. The largest increases in β-hydroxybutyrate were apparent by week 24 when most of the reduction in liver fat had occurred and percent changes in β-hydroxybutyrate and liver fat were significantly correlated. In a recent 24-week study, the dual GLP-1/GCG agonist, efinopegdutide, demonstrated greater liver fat lowering efficacy than semaglutide for the same degree of weight loss. For weight loss categories of ≤5%, >5% to ≤10%, and >10%, liver fat reduction with efinopegdutide (52.4%, 76.6% and 86.2%, respectively) was greater than with semaglutide (13.4%, 39.6% and 64.2%, respectively)^14^. These data suggest that glucagon agonism provides additional liver fat reducing efficacy beyond what is expected from weight loss alone. In this regard, it is noteworthy that a selective glucagon receptor antagonist increased hepatic fat in patients with T2D^23^. FGF21, a liver-secreted hormone, has been proposed as a potential mediator of GCG actions to regulate hepatic metabolism^24^. Previous studies have shown that chronic GCG receptor agonism increased circulating FGF21 in mice and acute glucagon administration transiently increased FGF21 levels in humans^25^. Notably, FGF21 analogs have reduced insulin resistance or decreased liver fat in clinical trials^16,26^; however, administration of retatrutide at higher doses significantly reduced circulating FGF21 levels, suggesting that retatrutide efficacy in MASLD is unlikely to be attributable to increased FGF21 levels.

Reductions in liver fat with retatrutide treatment were significantly related to changes in body weight, ASAT and VAT. A near-maximal liver fat reduction of approximately 75% was achieved coincident with an approximately 20% reduction in body weight. Thus, almost all the reduction in liver fat occurred within the first 24 weeks. In contrast, significant further reductions in ASAT and VAT continued beyond 24 weeks in parallel with continued weight loss. This finding is consistent with a ‘floor effect’ for hepatic fat, which is unlikely to be reduced below the median of 2.1% observed in the large UK Biobank study^27^. Therefore, with weight loss greater than 20%, the relative reduction of liver fat approaches an asymptote at the maximal achievable liver fat loss. These data also suggest that under conditions favoring fat mobilization and oxidation, hepatic fat is preferentially mobilized over fat from adipose tissue depots.

In addition to reductions in body weight, abdominal fat and liver fat, retatrutide treatment was associated with improvements in insulin sensitivity, lipid metabolism and adipocyte hormones. Higher doses of retatrutide reduced biomarkers of insulin resistance, including fasting insulin, fasting C-peptide and HOMA2-IR, by up to 50% or more from baseline. Time- and dose-related improvements in insulin sensitivity were similar to the temporal patterns of body weight loss with retatrutide in both the MASLD cohort reported here and in the full cohort of people with obesity^13^. Retatrutide-mediated reductions in markers of insulin resistance were closely associated with decreased liver fat. Retatrutide improved fasting lipid profiles including triglycerides, very low-density lipoprotein cholesterol and non-high-density lipoprotein cholesterol in clinical trials in people with T2D or obesity, while low-density lipoprotein cholesterol was reduced in people with obesity^12,13,28^. In the current analysis in people with MASLD, fasting triglycerides were reduced by greater than 40% by retatrutide 8 mg and 12 mg doses after 48 weeks, and these reductions were significantly associated with reduced liver fat. Activation of both GIP and GLP-1 receptors by retatrutide probably contributed to the observed changes in circulating triglycerides, as treatment with the dual GIP/GLP-1 receptor agonist tirzepatide significantly reduced triglyceride levels by up to 29% at 52 weeks in a clinical trial in people with T2D and MASLD^8^. Retatrutide also reduced liver triglyceride levels in preclinical studies of obese mice^11^. The observed reductions in leptin levels with higher doses of retatrutide may reflect a function of this hormone as a circulating indicator of fat stores^29^. Adiponectin levels increased in a time-related manner with higher doses of retatrutide, consistent with progressive adipose tissue reductions and improvements in insulin sensitivity^30^.

Several MASH-related biomarkers were evaluated in this study. Significant reductions were observed for K-18 with the 8 mg and 12 mg doses of retatrutide (up to 49.6%) and for pro-C3 with all doses of retatrutide (up to 26.4%). K-18 is released during hepatocyte apoptosis and has been proposed as a marker of cell death in the liver^31^. A recent meta-analysis proposed K-18 values between 127–191 U l^−1^ for ruling-out MASH at fixed sensitivity levels and 304–399 U l^−1^ to rule-in MASH at fixed specificity levels^32^. In this study, mean baseline K-18 levels ranged from 263 to 311 U l^−1^. The magnitude of decrease in K-18 observed with retatrutide 8 mg and 12 mg has been associated with greater odds of histological improvement in previous studies of patients with MASH^33^. Pro-C3 is an epitope that is generated during procollagen type III cleavage and reflects fibrogenic drive^34^. Plasma pro-C3 levels have been shown to correlate with severity of steatohepatitis and fibrosis stage in patients with MASLD^35^. In a longitudinal study of such patients, mean pro-C3 increased with worsening of fibrosis and decreased with fibrosis improvement^36^. Changes in pro-C3 have also been shown to correlate with changes in fibrosis stage in patients with T2D and MASLD after 18 months of treatment with pioglitazone, vitamin E or placebo^37^. FIB-4 and ELF score are two well-validated biomarkers that assess the risk for liver fibrosis in patients with MASLD. A FIB-4 index of <1.3 and an ELF score of <9.8 have both been used to rule-out advanced fibrosis^38,39^. In this study, mean baseline FIB-4 index ranged from 0.7 to 0.9 and mean baseline ELF score ranged from 7.8 to 8.3. Only seven participants (7.1%) had either a FIB-4 index higher than 1.3 or an ELF score higher than 9.8. Thus, it is likely that most participants in this study had simple steatosis or MASH with mild fibrosis. Therefore, significant improvements in FIB-4 and ELF would not be expected in this population. Mean ALT and AST levels were also normal at baseline; thus, the absence of consistent change in these enzymes after retatrutide treatment is neither surprising nor determinant. The reasons for the discrepancy between pro-C3 and ELF results are not entirely clear but may reflect the fact that the ELF test is a proprietary algorithm based on three fibrosis biomarkers (hyaluronic acid, procollagen III amino acid terminal peptide and tissue inhibitor of metalloproteinase 1), whereas pro-C3 is a single biomarker targeting the N-terminal pro-peptide of type III collagen by a different epitope than the procollagen III amino acid terminal peptide assay included in the ELF test. A potential explanation of the difference in response between the two tests is that pro-C3 may be more indicative of active fibrogenesis, whereas ELF may correlate more with the severity of histological liver fibrosis^36,40^.

The safety profile of retatrutide in people with MASLD was similar to that observed in the broader trial population of people with obesity^13,28^. Transient, mostly mild-to-moderate gastrointestinal events were the most frequently reported adverse events, occurring primarily during dose escalation. The frequency of these adverse events was higher in the 8 mg and 12 mg dose groups than in the other dose groups. There were no hepatotoxicity signals in the overall obesity trial population or in the subset of participants with MASLD through 48 weeks. These safety findings are similar to those reported for therapies based on GLP-1 or GIP/GLP-1 agonism for the treatment of type 2 diabetes or obesity^41–45^. This study did not include patients with advanced fibrosis or cirrhosis; thus, potential safety concerns with use of retatrutide in such patients cannot be assessed from these data.

The strengths of this trial include its prospective design with a priori defined endpoints, the inclusion of approximately equal percentages of men and women and 41.8% of participants identifying as Hispanic or Latino, the evaluation of liver fat and body composition by MRI and the measurement of several biomarkers to indirectly assess progressive MASLD and suggest potential mechanistic insights. The limitations include the relatively small sample size of the MASLD substudy, the geographic and racial homogeneity of the sample (United States only and majority white), the exclusion of patients with T2D, the absence of liver histology, the lack of enrichment for MASH or significant fibrosis, the lack of multiplicity control given the large number of statistical assessments and the absence of 48-week MRI data for 56.1% of participants, which limits interpretation of the dose-response relationship for liver fat reduction at 48 weeks. However, despite this latter limitation, the 48-week data demonstrate that the defatting of the liver that occurred by 24 weeks was maintained at 48 weeks. The results of this phase 2 substudy should be considered as hypothesis-generating and not definitive.

In this phase 2 trial in participants with obesity and MASLD, once-weekly treatment with the GIP/GLP-1/GCG triple agonist retatrutide resulted in substantial reductions in liver fat, body weight, ASAT and VAT, which were associated with improvements in insulin sensitivity, serum lipids, K-18 and pro-C3. Hepatic steatosis resolved in more than 85% of participants in the two highest dose groups. These results suggest that retatrutide may be an effective therapeutic agent for treatment of MASLD. Further studies are warranted to determine whether retatrutide treatment can reduce the severity of fibrotic MASH and reduce the risk of major adverse liver outcomes in people with MASLD.

## Methods

### Study design and participants

The main study (NCT04881760) was a 48-week, phase 2, multicenter, randomized, double-blind, placebo-controlled study designed to examine the safety and efficacy of retatrutide, administered subcutaneously once weekly in participants with obesity (BMI of 30 kg m^−^^2^ or greater), or overweight (BMI ≥27 and <30 kg m^−^^2^) with weight-related complications other than T2D. The study sites (all in the United States) and the inclusion and exclusion criteria for the main study were previously reported^13^. A substudy of the main study, presented here, enrolled participants who qualified for the main study and also had MASLD with a liver fat content of 10% or greater identified by MRI–PDFF. For the substudy, additional exclusion criteria included contraindication to MRI examinations and claustrophobia precluding completion of an MRI examination. To achieve the enrollment target for the substudy (approximately 100 participants), the main study was overenrolled (*n* = 338 versus planned *n* = 300).

The trial was approved by the institutional review board or ethics committee at each site. The study was conducted in accordance with the consensus ethical principles from the Declaration of Helsinki and Council for International Organizations of Medical Sciences International Ethical Guidelines and International Council for Harmonisation of Technical Requirements for Pharmaceuticals for Human Use. Participants provided written, informed consent. The study sponsor designed and executed the trial. All authors had access to trial data and were involved in the preparation of the paper.

### Randomization and masking

In the main study, participants were enrolled by study investigators and randomly assigned in a 2:1:1:1:1:2:2 ratio (with stratification according to sex, BMI (<36 or ≥36 kg m^−^^2^) and substudy participation) using an interactive web response system to receive once-weekly injections of retatrutide 1 mg, retatrutide 4 mg (2 mg starting dose), retatrutide 4 mg (4 mg starting dose), retatrutide 8 mg (2 mg starting dose), retatrutide 8 mg (4 mg starting dose), retatrutide 12 mg (2 mg starting dose) or placebo once weekly. The dose-escalation scheme is illustrated in Extended Data Fig. 1. An upper limit of 60% enrollment of women was used to ensure a sufficiently large sample of men. Retatrutide and placebo were provided in matching single-use vials. As the substudy included only 29% of the sample of the main study (98 of 338 participants), the starting dose subgroups for the 4 mg and 8 mg treatment arms were pooled for analyses in this substudy.

### Procedures and assessments

The study consisted of three periods: a 6 week screening period, a 48 week treatment period and a 4 week safety follow-up (Extended Data Fig. 1). A machine learning algorithm using XGBoost^46^, developed with data from previous Lilly clinical trials, based on BMI, sex, fasting ALT, fasting AST and fasting triglycerides was used to identify study participants at increased probability for liver fat content of 10% or greater who would then undergo liver fat assessment by MRI–PDFF to determine eligibility for the substudy. MRIs were performed at imaging centers near the investigators’ sites after a fast of at least 6 h at baseline, week 24 and week 48. The allowable interval tolerance for the MRI was ±14 days. In the event of an early discontinuation from study treatment, the early discontinuation visit included an MRI assessment if the participant had been on study treatment for at least 16 weeks; this MRI was specified to be done within 2 weeks of the last dose of study drug.

The MRI acquisition protocol included sequences for measurement of liver fat content and abdominal fat (including VAT and ASAT volumes). Scans were done at 1.5 T or 3 T at each site (using MRI scanners manufactured by Siemens, Philips or General Electric). The same scanner and imaging acquisition parameters were used for both baseline and postbaseline time points for each participant. To obtain consistent and quality data for central review by a qualified vendor (BioTel Research; Cardiocore and VirtualScopics), investigators participating in the substudy were provided with a standardized imaging acquisition protocol. Liver fat content was assessed with MRI–PDFF and was expressed as the mean fat fraction across all nine user-defined regions of interest in the liver. The average value of the nine regions of interest was analyzed as the mean hepatic fat fraction. The regions of interest were defined on the shortest time to echo image of each slice. VAT and ASAT were assessed with body composition analyses by AMRA Profiler Research (AMRA Medical AB). VAT analysis consisted of the measurement of the adipose tissue within the abdominal cavity, excluding adipose tissue outside the abdominal skeletal muscles and adipose tissue and lipids within the cavity and posterior of the spine and back muscles. ASAT analysis consisted of the measurement of the subcutaneous adipose tissue in the abdomen from the top of the femoral head to the top of the thoracic vertebra T9. The details for the MRI sequence parameters and postprocessing were the same as previously reported except, for this study, the phantom belt consisted of five lipid phantom vials containing liquid with fat fractions of 0%, 10%, 20%, 30% and 40% (by volume)^8^.

Serum biomarkers of MASH and fibrosis were also collected at baseline, week 24 and week 48 or at the time of an early discontinuation. These included measurement of ALT, AST, FIB-4 index, K-18, ELF panel and pro-C3 (a fragment of the NH_2_-terminal pro-peptide of type III procollagen). In addition, metabolic biomarkers related to insulin sensitivity, lipid storage and metabolism were assessed. These included measurement of fasting insulin, C-peptide, triglycerides, adiponectin, leptin, beta-hydroxybutyrate, free fatty acids and FGF21.

Safety and tolerability were assessed at all time points and during follow-up. All participants received diet and physical activity counseling using a standardized approach throughout the study, as previously described^13^.

### Study outcomes

The primary objective of the substudy was to assess retatrutide doses of 1 mg, 4 mg, 8 mg and 12 mg compared with placebo at week 24 for relative liver fat change measured by MRI–PDFF. Secondary outcomes included the effect of retatrutide treatment at week 48 compared with placebo for relative liver fat change, absolute liver fat change at weeks 24 and 48, and the percentage of participants achieving a 30% or greater relative liver fat reduction. Exploratory analyses included the percentage of participants achieving 50% or greater relative and absolute liver fat reduction and liver content lower than 5%. Other exploratory objectives included retatrutide effects on VAT volume, ASAT volume, serum metabolic biomarkers and biomarkers of MASH and fibrosis compared with placebo.

### Statistical analysis

The sample size for the substudy was calculated to ensure a power of at least 80% for detecting the superiority of any dose (1, 4, 8 or 12 mg) of retatrutide versus placebo in change in relative liver fat by MRI–PDFF from baseline to week 24. Assuming a treatment effect of 30%, a standard deviation of 27.28%, a two-sided *t*-test with an *α* level of 0.05 and a 20% dropout rate for retatrutide, it was estimated a total sample size of 100 randomized participants was needed (that is, 20 participants per group). The starting dose subgroups for the 4 mg and 8 mg treatment arms were pooled to provide enough statistical power within the constraints of a relatively limited sample size. All tests of treatment effect were performed at a two-sided significance level of 0.05 and two-sided 95% confidence intervals. No multiplicity adjustments were made.

All efficacy analyses were guided by efficacy estimand and conducted on the efficacy analysis set with all randomized substudy participants. Data after intercurrent events (for example, permanent treatment discontinuation) were excluded. Safety analyses were conducted on the safety analysis set with all randomized substudy participants who received at least one dose of study drug. Analyses on continuous endpoints were conducted using a mixed model for repeated measures with treatment, visit, stratification factors and treatment by visit, stratification factors by visit and baseline measurement by visit interactions as fixed effects, baseline measurement as a covariate and participant as a random effect. No imputation was considered for missing data. Analyses on binary endpoints were conducted using a logistic regression with treatment and stratification factors as fixed effects and baseline measurement as a covariate. For missing binary response of liver fat content, continuous liver fat data at the scheduled visit were imputed using the efficacy analysis set assuming missing at random with multiple imputation. The imputed continuous values were then transformed into binary response, which was used in logistic regression^47^. Rubin’s Rule was used for the final inference by combining estimates from imputed datasets. Correlations were evaluated with either Spearman correlation or a power model with efficacy analysis set without imputation of missing data.

Statistical analyses were computed using statistical software R (version 4.2.2). This is a substudy of the trial registered with ClinicalTrials.gov, number NCT04881760.

### Role of funding source

The funder of the study provided study drugs and was involved in study design, data collection, data analyses, data interpretation and writing of the report.

### Reporting summary

Further information on research design is available in the Nature Portfolio Reporting Summary linked to this article.

## Online content

Any methods, additional references, Nature Portfolio reporting summaries, source data, extended data, supplementary information, acknowledgements, peer review information; details of author contributions and competing interests; and statements of data and code availability are available at 10.1038/s41591-024-03018-2.

### Supplementary information

Reporting Summary

## Figures and Tables

**Extended Data Table 1:**
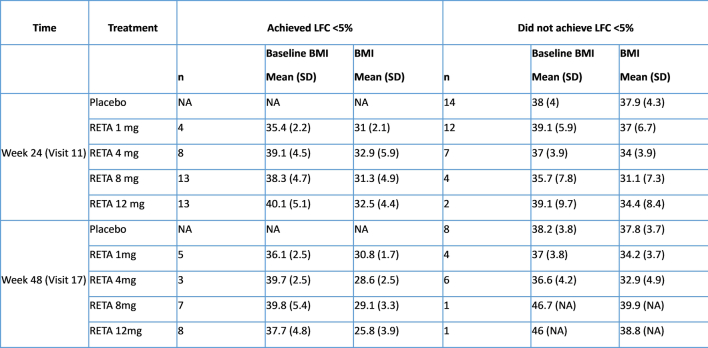
BMI for participants achieving or not achieving liver fat content <5% at weeks 24 and 48

BMI – body mass index; LFC – liver fat content; RETA – retatrutide; SD – standard deviation.

**Extended Data Table 2:**
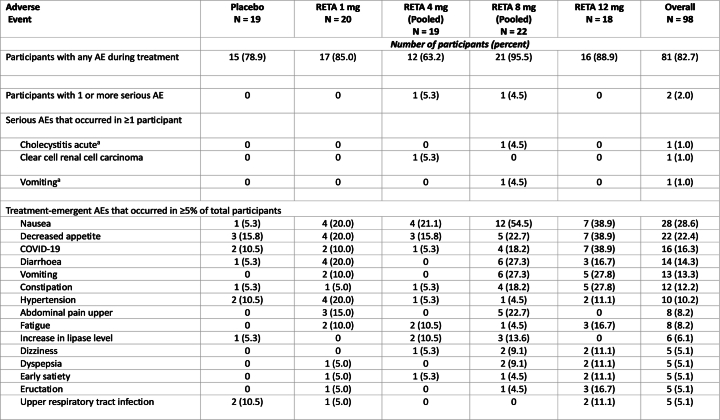
Adverse events and safety

AE, adverse event; RETA, retatrutide. Safety endpoints were analyzed with data from all substudy participants who underwent randomization and received at least one dose of retatrutide or placebo. ^a^One participant had serious adverse events of cholecystitis acute and vomiting; this participant received intravenous hydration and a laparoscopic cholecystectomy was performed.

## Data Availability

Lilly provides access to all individual participant data collected during the trial, after anonymization, with the exception of pharmacokinetic or genetic data. Data are available for request 6 months after the indication studied has been approved in the United States and European Union and after primary publication acceptance, whichever is later. No expiration date of data requests is currently set once data are made available. Access is provided after a proposal has been approved by an independent review committee identified for this purpose and after receipt of a signed data sharing agreement. Data and documents, including the study protocol, statistical analysis plan, clinical study report and blank or annotated case report forms, will be provided in a secure data sharing environment. For details on submitting a request, see the instructions provided at www.vivli.org.
